# Supplementary material for: Conditional chemoconnectomics (cCCTomics) as a strategy for efficient and conditional targeting of chemical transmission
Source: eLife. 2024 Apr 30;12:RP91927. doi: 10.7554/eLife.91927 (PMC11060718; doi:10.7554/eLife.91927)
Supplement: Figure 3—source data 3. [file elife-91927-fig3-data3.zip › Figure 3 source data3 part 2.zip!/Figure 3-Source data 3-part2/1052-13-CRISPResso_Report_ejedac2.zip!/CRISPResso_on_1052_16/3b.Insertion_deletion_substitutions_size_hist.pdf]

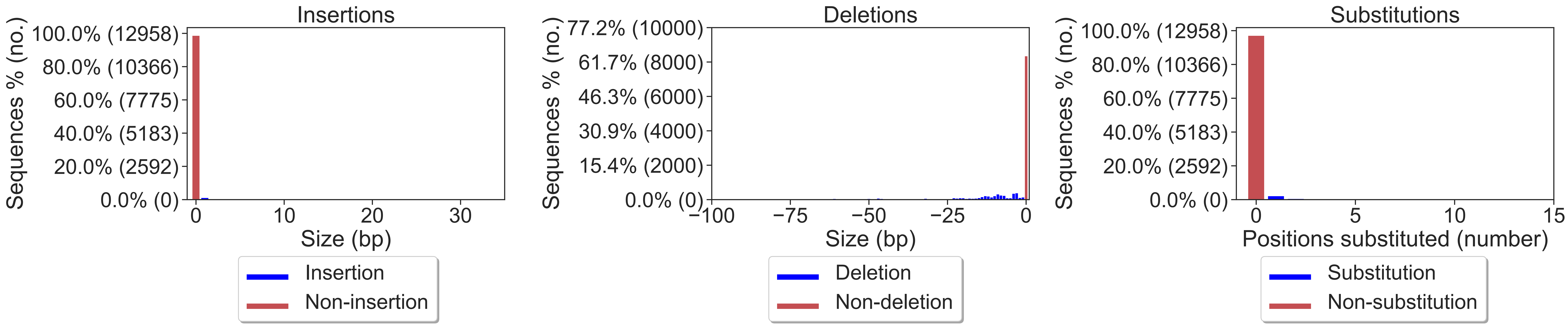

Insertions
Sequences % (no.)
100.0% (12958)
80.0% (10366)
60.0% (7775)
40.0% (5183)
20.0% (2592)
0.0% (0)
0
10
20
30
Size (bp)
Insertion
Non-insertion
Deletions
Sequences % (no.)
77.2% (10000)
61.7% (8000)
46.3% (6000)
30.9% (4000)
15.4% (2000)
0.0% (0)
−100
−75
−50
−25
0
Size (bp)
Deletion
Non-deletion
Substitutions
Sequences % (no.)
100.0% (12958)
80.0% (10366)
60.0% (7775)
40.0% (5183)
20.0% (2592)
0.0% (0)
0
5
10
15
Positions substituted (number)
Substitution
Non-substitution
